# Präoperative Therapie des Rektumkarzinoms – Kritische Anmerkungen zur amerikanischen PROSPECT-Studie

**DOI:** 10.1007/s00066-023-02116-3

**Published:** 2023-07-10

**Authors:** Emmanouil Fokas, Claus Rödel

**Affiliations:** Klinik für Strahlentherapie, Universitätsklinikum Frankfurt, Goethe Universität Frankfurt, Frankfurt, Deutschland

## Hintergrund

Die amerikanische *NCCN Guideline* zur Therapie des Rektumkarzinoms empfiehlt derzeit für Patientinnen und Patienten mit sogenanntem „*locally advanced rectal cancer*“ (cT1–2 N1–2, cT3–4 N0–2) die präoperative Radiotherapie (RT) oder Radiochemotherapie (RCT) im Rahmen der totalen neoadjuvanten Therapie (TNT), gefolgt von totaler mesorektaler Exzision (TME). Die PROSPECT-Studie untersuchte, ob eine neoadjuvante Chemotherapie mit nur selektivem Einsatz der RCT einer generellen RCT bezüglich des krankheitsfreien Überlebens für eine Subgruppe des Rektumkarzinoms nicht unterlegen ist.

## Methoden

Eingeschlossen waren Patientinnen und Patienten mit niedrigem Lokalrezidivrisiko (cT2 N1, cT3 N0/1, radialer Abstand zur mesorektalen Faszie > 3mm), die sich aufgrund der Tumorlage für eine schließmuskelerhaltende Operation eigneten. Fortgeschrittene Tumorcharakteristika (T4, N2, Abstand zur mesorektalen Faszie ≦ 3 mm, tief sitzende Tumoren, bei denen der Schließmuskel operativ nicht erhalten werden konnte) waren ausgeschlossen. Die Randomisierung erfolgte in einen Arm mit neoadjuvanter Chemotherapie (6 Zyklen FOLFOX) mit nur selektivem Einsatz der RCT (bei < 20% Tumoransprechen oder < 5 Zyklen FOLFOX) und einen Arm mit genereller neoadjuvanter RCT (50,4 Gy in 28 Fraktionen, simultan 5‑FU/Capecitabin). Nach obligat erfolgter radikaler Operation (kein „watch and wait“ vorgesehen) war die weitere Therapie freigestellt. Empfohlen war von der Studienleitung jedoch die adjuvante Chemotherapie mit FOLFOX (6 bzw. 8 Zyklen nach neoadjuvant FOLFOX bzw. RCT) für alle Patientinnen und Patienten unabhängig vom histopathologischen Tumorstadium. Der primäre Endpunkt war die Nichtunterlegenheit in Bezug auf das krankheitsfreie Überleben (Hazard Ratio für Rezidiv oder Tod < 1,29).

## Ergebnisse

Von Juni 2012 bis Dezember 2018 wurden 1194 Patientinnen und Patienten randomisiert; 1128 erhielten eine neoadjuvante Therapie (585 in der FOLFOX-Gruppe und 543 in der RCT-Gruppe). Bei einer medianen Nachbeobachtungszeit von 58 Monaten führten beide Therapiearme zu einer exzellenten lokalen Tumorkontrolle (< 2 % Lokalrezidive) sowie einem nahezu identischen krankheitsfreien Überleben (HR 0,92; 90,2 %-KI 0,74–1,14; *p* = 0,005 für Nichtunterlegenheit) und Gesamtüberleben (89,5 % versus 90,2 % nach 5 Jahren). Unterschiede traten allerdings in Art und Ausprägung von Nebenwirkungen auf. Diese waren unter neoadjuvanter FOLFOX-Chemotherapie deutlich ausgeprägter (Grad 3–4: 41 % versus 22,8 % unter neoadjuvanter RCT) und betrafen insbesondere die Neutropenie sowie Neuropathie, während eine Diarrhö häufiger im RCT-Arm auftrat.

In einer Begleitpublikation zu PROM berichteten die Patientinnen und Patienten während der Therapie sowie 1 Jahr nach Operation von einem unterschiedlichen Beschwerdebild: Unter neoadjuvanter Chemotherapie waren u. a. Müdigkeit, Appetitlosigkeit, Übelkeit/Erbrechen, Sensibilitätsstörungen und Depression/Ängstlichkeit ausgeprägter, während Durchfall häufiger bei der neoadjuvanten RCT berichtet wurde. Ein Jahr nach Operation waren in beiden Armen die akut beklagten Beschwerden rückläufig und keine Symptome wurden (von > 15 % der Patientinnen und Patienten) mehr als „ausgeprägt“ beschrieben. Bezüglich Fatigue, Neuropathie und Sexualfunktion berichteten bestrahlte Patientinnen und Patienten zu diesem Zeitpunkt über mehr Beschwerden. Die generelle Lebensqualität (HRQL) war in beiden Armen 1 Jahr nach Operation identisch. Daten zu Langzeitnebenwirkungen liegen noch nicht vor.

## Schlussfolgerung der Autoren

Für Patientinnen und Patienten mit den oben beschriebenen Tumorcharakteristika war die präoperative FOLFOX-Chemotherapie der präoperativen RCT in Bezug auf das krankheitsfreie Überleben nicht unterlegen (ClinicalTrials.gov, NCT01515787).

## Kommentar

Die PROSPECT-Studie wurde vor mehr als 12 Jahren konzipiert und berücksichtigte wesentliche moderne Aspekte der multimodalen Behandlung des Rektumkarzinoms nicht. In Europäischen Leitlinien und der Deutschen S3-Leitlinie ist längst verankert, dass bei Patientinnen und Patienten mit a priori niedrigem Lokalrezidivrisiko, wie sie in der PROSPECT-Studie überwiegend eingeschlossen waren, die primäre Operation mit stadiengerechter adjuvanter Chemotherapie eine valide Therapiealternative ist. Insofern stellen aus deutscher und europäischer Sicht beide neoadjuvanten Therapiegruppen der PROSPECT-Studie eine nicht unerhebliche, potenzielle Übertherapie dar (mit einer Grad-3- bis Grad-4-Akuttoxizität von 41 % und 23 % bei neoadjuvantem FOLFOX bzw. RCT). Dies trifft auch für die zwar nicht obligat vorgegebene, aber überwiegend durchgeführte adjuvante Chemotherapie zu, deren „benefit“ nach erfolgter neoadjuvanter Therapie in Phase-3-Studien und Metaanalysen nicht belegt ist.

Aus Sicht der Radioonkologie ist überdies kritisch anzumerken, dass ein MRT des Beckens nicht obligat gefordert war, dass die radiotherapeutische Technik (3-D-konformal versus IMRT) freigestellt war (und nicht berichtet wird), dass die im Protokoll vorgegebene Definition der Zielvolumina moderne Entwicklungen nicht berücksichtigt und dass 20–22 % der Patientinnen und Patienten Tumoren im oberen Drittel und darüber hinaus (bis 25 cm ab Anokutanlinie!) aufwiesen. Methodisch sollte auch berücksichtigt werden, dass die PROM zur Sexualfunktion 1 Jahr nach Operation im RCT-Arm auf nur 34/173 Patientinnen (20 %) bzw. 97/370 Patienten (26 %) beruhen.

Die laufende ACO/ARO/AIO-18.2-Studie der *German Rectal Cancer Study Group* vergleicht derzeit bei weitgehend mit PROSPECT überlappenden Einschlusskriterien konsequenterweise im Kontrollarm die primäre TME gefolgt von stadiengerechter adjuvanter Chemotherapie (nur bei pT4 oder pN+) mit einer generellen neoadjuvanten FOLFOX/CapOx-Chemotherapie über 3 Monate. Eine adjuvante Chemotherapie nach TME ist in diesem experimentellen Arm nicht vorgesehen. Das primäre Ziel dieser Studie ist es, eine Überlegenheit bezüglich des DFS zu zeigen. Um den Stellenwert der neoadjuvanten Chemotherapie in einem Patientenkollektiv mit niedrigem Lokalrezidivrisiko besser einordnen zu können, sollten nach Meinung der Kommentatoren die Ergebnisse dieser deutschen Studie dringend abgewartet werden.

Darüber hinaus haben moderne radioonkologische Strategien zur Behandlung des Rektumkarzinoms in den zurückliegenden 10 Jahren sehr erfolgreich das Konzept des Funktions- und Organerhalts entwickelt. Dabei wird abhängig vom Therapieansprechen auf eine Radiochemotherapie mit oder ohne neoadjuvante Chemotherapie (sogenannte totale neoadjuvante Therapie) auf die radikale Operation verzichtet und die Patientinnen und Patienten in ein Watch-and-wait-Programm überführt. Daten aus aktuellen Studien zeigen, dass diese Strategie je nach Tumorstadium bzw. Tumorgröße in 50 % bis 80 % zu einem langfristigen Organerhalt ohne Operation bei sehr gutem Funktionserhalt und sehr guter Lebensqualität führen kann [1–3].

### Fazit

Die PROSPECT-Studie erweitert prinzipiell die Therapieoptionen der multimodalen Behandlung des Rektumkarzinoms für eine klinisch und bildgebend definierte Subgruppe mit a priori niedrigem Lokalrezidivrisiko, stellt aber eine nicht unerhebliche potenzielle Übertherapie dar. Im Gesamtkontext aller verfügbaren Evidenz stehen für dieses Patientenkollektiv mehrere Therapiealternativen zur Verfügung. Die Beratung zu diesen Optionen im Sinne eines „*shared decision-making“* sollte unter obligater Einbindung aller beteiligten Fachdisziplinen auf dem Boden einer qualitätsgesicherten MRT-Untersuchung und einer MSI-Untersuchung erfolgen:

- Primäre TME gefolgt von stadiengerechter adjuvanter Chemotherapie in Analogie zum Kolonkarzinom
- Primäre RT/RCT gefolgt von TME oder W&W bei klinischer Komplettremission
- Neoadjuvante FOLFOX/CapOx-Chemotherapie gefolgt von obligater TME
- Totale neoadjuvante Therapie analog OPRA [1] oder anderen etablierten TNT-Protokollen mit W&W bei explizit gewünschtem und intendiertem Organerhalt
- Primäre RCT + Brachytherapie analog OPERA [2] mit W&W bei explizit gewünschtem und intendiertem Organerhalt
- Definitive Immuntherapie über 6 Monate bei Nachweis einer MSI/dMMR [4]

*Emmanouil Fokas, Claus Rödel, Frankfurt*
